# Real-world predictors of changes in fear of COVID-19 in the Japanese general population: a large-scale internet-based cohort study with 20,712 participants

**DOI:** 10.1186/s12888-024-05899-6

**Published:** 2024-06-11

**Authors:** Keita Tokumitsu, Norio Sugawara, Takahiro Tabuchi, Norio Yasui-Furukori

**Affiliations:** 1 Department of Neuropsychiatry, Towada City Hospital, Towada, Aomori, Japan; 2 Department of Psychiatry, Dokkyo Medical University School of Medicine, Mibu, Tochigi, Japan; 3 Cancer Control Center, Osaka International Cancer Institute, Osaka, Japan; 4 Tokyo Foundation for Policy Research, Tokyo, Japan

**Keywords:** COVID-19, Fear of COVID-19 Scale, Predictor, Japanese

## Abstract

**Background:**

Coronavirus disease 2019 (COVID-19) is a respiratory infection that considerably impacts both physical and mental health. In particular, the prolonged nature of psychological issues associated with COVID-19 has become a concern. However, evidence based on longitudinal studies investigating the changes in fear of COVID-19 has been limited, posing a public health challenge.

**Methods:**

We investigated the predictors of changes in the Fear of COVID-19 Scale (FCV-19S) scores in the general Japanese population using data from a large-scale internet-based cohort study.

**Results:**

We included 20,712 study participants (mean age = 51.1 years, percentage of males = 49.9%). The baseline FCV-19S score for the research participants was 17.0, and one year later, the FCV-19S score decreased to 15.8. The predictors of increase in FCV-19S scores were older age, male sex, COVID-19 requiring oxygen therapy, higher baseline FCV-19S total score, severe psychological distress, never married, worsening subjective health status, a greater number of COVID-19 vaccinations, a history of alcohol dependency, and living with family members. Conversely, the predictors of decrease in FCV-19S scores included habitual alcohol intake, COVID-19 not requiring oxygen therapy and a higher household income. Our study was an internet-based survey focused on residents of Japan, which raises the possibility of selection bias and makes it unclear whether the findings can be extrapolated to other countries and cultures.

**Conclusion:**

During the observation period, the FCV-19S scores significantly decreased. Severe COVID-19 requiring oxygen therapy within one year of baseline was the most impactful predictor of an increase in FCV-19S score. On the other hand, mild COVID-19 not requiring oxygen therapy was a predictor of a decrease in FCV-19S scores. Therefore, we believe that it is necessary to adopt individualized approaches stratified by the severity of the infection when addressing the fear of COVID-19.

**Supplementary Information:**

The online version contains supplementary material available at 10.1186/s12888-024-05899-6.

## Background

Since the identification of coronavirus disease 2019 (COVID-19) in December 2019, the number of related deaths has continued to increase [1]. In the initial stages, the focus was on managing and treating physical conditions; however, gradually, mental health issues became more apparent [2]. Notably, with the increasing risk of COVID-19, there has been an increase in concerns related to anxiety and fear of COVID-19 [3]. Fear of the virus mutating, anxiety about an unknown illness, discrimination and prejudice due to COVID-19, and social isolation due to restrictive measures all imposed considerable psychological burdens on individuals [3]. Furthermore, the COVID-19 pandemic severely impacted the socioeconomic structure, leading to dysfunction [4]. Owing to these psychosocial stressors, there has been an increase in mental health disorders, including depression, anxiety and adjustment disorders during the COVID-19 pandemic regardless of region or age [5, 6]. Some cases escalate to the point of trauma, leading to long-term psychological consequences [7, 8].

In general, while many infections have acute and transient impacts, the psychological effects of COVID-19 persist, leading to heightened levels of fear and anxiety. In the early stages of the COVID-19 pandemic, mental health support was provided for healthcare workers involved in treatment of individuals infected with COVID-19 [9]. However, recently, stress responses in the general population during the pandemic have become a concern, irrespective of infection status, and research is underway on public health strategies [10]. Understanding the predictors of fear during the COVID-19 pandemic in the general population is valuable not only for academic pursuits but also for assisting in effective public health interventions and targeted mental health support. Previous studies have conducted cross-sectional surveys on factors related to fear during the COVID-19 pandemic [11–13]. However, there is insufficient evidence to assess the predictors of fear of COVID-19.

The primary objective of this study was to elucidate the predictors of changes in the Fear of COVID-19 Scale (FCV-19S) in the general Japanese population and to provide valuable insights into the factors influencing the trajectory of fear during the ongoing pandemic. We utilized data from a large-scale internet-based cohort study known as the Japan Society and New Tobacco Internet Survey (JASTIS), initiated in 2015 to explore the social impact of heated tobacco products [14, 15]. Following the onset of the COVID-19 pandemic, additional questions related to the impact of COVID-19 were incorporated, and annual surveys were performed. Therefore, the data obtained from the JASTIS are instrumental in investigating the predictors of fear in the general population during the COVID-19 pandemic. The FCV-19S was designed to measure individual levels of fear related to COVID-19 and served as a crucial tool in this survey [3, 16]. Building upon these societal backgrounds and research needs, we investigated the predictors of changes in fear during the COVID-19 pandemic in the general Japanese population using a longitudinal approach with the FCV-19S.

## Methods

### Study design and participants

This study used data from the large-scale online JASTIS [14]. JASTIS is a longitudinal study project that comprises a series of annual online surveys that have been undertaken since 2015. As of June 1, 2022, a total of eight survey waves have been conducted. In this study, we used data from the survey of the 2022 JASTIS, which was conducted from the 1st to 28th of February 2022, and from the 2023 JASTIS, which was conducted from the 6th to 27th of February 2023. Details of the JASTIS have been previously published [14].

Among the 33,000 respondents of JASTIS 2022, we initially screened all respondents and excluded individuals who met any of the following three criteria [17]: (1) Those who selected other than the second from the bottom in the question of “please select the second option from the bottom out of five options (A, B, C, D, and E)”; (2) Those who selected “yes” to all drug use (do you use the following drugs? alcohol, strong chu-hi with an alcohol content of 9% or more, sleeping pills/antianxiety medications, morphine, thinner, marijuana, dangerous drugs [e.g., outlawed herbs, magic mushrooms, etc.], and cocaine/husk [eight items]); (3) Those who selected “yes” to comorbidity of all diseases (do you have the following diseases? hypertension, diabetes, asthma, atopic dermatitis, allergic rhinitis, angina pectoris/myocardial infarction, stroke [cerebral infarction or cerebral hemorrhage], cancer/malignant tumor, and chronic pain [nine items]).

We excluded participants who had history of COVID-19 before baseline (JASTIS 2022). Finally, we excluded individuals who did not participate in JASTIS 2023.

### Outcome measurement

We used the Japanese version of the FCV-19S. The FCV-19S is a brief self-report instrument that assesses an individual's level of fear of COVID-19 [3, 13]. It is frequently employed in both research and clinical environments to promptly recognize individuals who might be experiencing feelings of fear. It comprises seven items (e.g., ‘I am most afraid of Coronavirus‐19’), scored on a five‐item Likert‐type scale ranging from 1 (strongly disagree) to 5 (strongly agree). The scale scores range from 7 (minimum) to 35 (maximum), with higher scores indicating greater fear of COVID‐19. The validity and reliability of the Japanese version of the FCV-19S have been confirmed [16].

### Exposure to COVID-19

Respondents were classified into three groups based on their exposure to COVID-19 during the follow-up period: those who were infected with COVID-19 and required oxygen therapy, those who were infected with COVID-19 but did not require oxygen therapy, and those who were not infected with COVID-19. Until May 8, 2023, when the classification of COVID-19 under the Infectious Disease Act was changed from a novel influenza infection to a class V infectious disease, all newly identified COVID-19 cases in Japan were required to be identified through the Notifiable Disease Surveillance System, and confirmation was primarily based on laboratory tests, primarily nucleic acid amplification tests.

### Covariates

A broad set of 16 covariates in JASTIS 2022 that could be associated with the FCV-19S included the following: (1) sex, (2) age, (3) number of family members (1, 2, 3 or more), (4) marital status (married, never married, widowed, divorced), (5) employment (employer/self-employed, employee, student, homemaker, retired, unemployed), (6) education (high school or lower, college or higher), (7) habitual alcohol intake (not at all, 1 or fewer times per week, 2 or more times per week), (8) smoking status (nonsmoker, current smoker, former smoker), (9) home ownership, (10) number of COVID-19 vaccinations, (11) the Kessler Screening Scale for Psychological Distress (K6) score [18, 19], (12) adverse childhood experiences [20, 21] (0, 1 or more), (13) subjective health status (average, good, poor), (14) history of alcohol dependency, (15) history of depression, and (16) household income (Japanese yen (income < 4 million, 4 million ≤ income < 8 million, 8 million ≤ income, did not know/did not want to answer)). Although individuals with a missing value for any covariate were excluded from the regression analyses, a missing value category for income was used as a dummy variable (Did not know/Did not want to answer) because of the relatively large sample size.

### Statistical analyses

The longitudinal approach is well suited for examining changes in fear of COVID-19. However, there is a possibility that participants may be lost to follow-up, potentially introducing bias into the results [22]. To address nonrandom nonresponses, we applied inverse probability weighting (IPW) to the remaining participants in each survey by modeling the probability of not dropping out from the study [23]. Given that considerable differences in baseline characteristics were observed between responders and nonresponders, we established a logistic regression model to account for nonresponsiveness [23]. This model incorporated potential confounding factors, including a nonresponse variable. The nonresponse variable was regarded as a key predictor of nonparticipation in the subsequent survey [24]. We conducted a multivariate logistic regression analysis to calculate propensity scores, which were used to predict the probability of completing follow-up. In our models, all 16 covariates and participation in JACSIS 2022 or not were included as independent variables.

For the JASTIS study, invitations for JASTIS 2023 were primarily sent to JASTIS 2022 participants. Additionally, invitations were extended to former Japan COVID-19 and Society Internet Survey (JACSIS) [25] participants due to the similarities in the JACSIS and JASTIS sample selection processes, with the aim of achieving the designated sample size. Participation data from JACSIS 2022 were not used in subsequent analyses. All the questionnaires were designed such that the respondents had to answer each question before they could proceed to the next question, thereby precluding the issue of missing data. The inclusion of additional covariates that could predict follow-up participation in the subsequent survey may effectively make the “assumption of missing at random” appropriate in the process of propensity score calculation [26].

The demographic and clinical characteristics of the respondents are presented as proportions for nominal variables and mean values with standard deviations for continuous variables. A paired t test was subsequently conducted to assess the statistical significance of the difference in means between the baseline and one-year FCV-19S scores. Multiple regression analysis with IPW was used to calculate the estimate, standard error, t value and p value for the predictors of changes in the FCV-19S total score. In the analysis, COVID-19 exposure, baseline FCV-19S total score and all 16 covariates were used as independent variables, while the difference between the baseline and one-year FCV-19S scores (“one-year FCV-19S total score” – “baseline FCV-19S total score”) for each study participant was used as the dependent variable. Probability values for the statistical tests were two-tailed, and *p* < 0.05 was considered to indicate statistical significance.

All the statistical analyses were performed with IBM SPSS Statistics (IBM Corporation, Version 28.0.0.0) and EZR (Saitama Medical Center, Jichi Medical University, Saitama, Japan) [27]. EZR provides a graphical user interface for R (The R Foundation for Statistical Computing, Vienna, Austria, version 4.0.3). More precisely, EZR is a modified version of R Commander (version 2.7–1) that incorporates the statistical functions frequently used in biostatistics.

### Ethical considerations

All participants responded to the online questionnaires after they provided online informed consent, and their intention to participate in this survey was confirmed. A credit point known as an “E-point,” which can be used for online shopping or converted to cash, was provided to the participants as an incentive. All procedures were conducted in accordance with the ethical standards of the Declaration of Helsinki. This study was approved by the Institutional Review Board of the Osaka International Cancer Institute (approval number: 20084).

## Results

**Fig. 1:**
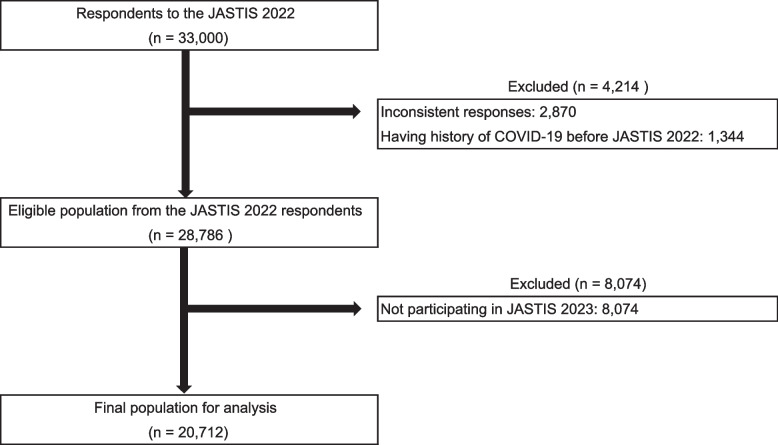
Participant flow chart. In JASTIS 2022, 28,786 participants met the eligibility criteria; however, in JASTIS 2023, 8,074 participants dropped out. A total of 20,712 individuals were included in the final analysis

In JASTIS 2022, 28,786 participants met the eligibility criteria; however, in JASTIS 2023, 8,074 participants dropped out. A total of 20,712 individuals were included in the final analysis (Fig. 1). The demographic characteristics of the study participants are presented in Table 1. The mean age of the research participants was 51.07 years, and the participants were 49.9% male and 50.1% female. The mean baseline FCV-19S total score was 17.01 (standard deviation = 5.49), and the mean FCV-19S total score one year later was 15.81 (standard deviation = 5.52). The results of the paired t test indicated a statistically significant difference in the mean FCV-19S score between baseline and one year later (*p* < 0.001).

**Table 1:** Clinical and demographic characteristics of study participants in final population for analysis

Factor	Group	Overall
n		20712
Age (mean (SD))		51.07 (16.91)
Sex (%)	female	10379 (50.1)
	male	10333 (49.9)
Living status (%)	Living alone	4485 (21.7)
	Living with family member	16227 (78.3)
Marital status (%)	Married	12525 (60.5)
	Never married	6117 (29.5)
	Widowed	722 ( 3.5)
	Divorced	1348 ( 6.5)
Employment (%)	Employee	11282 (54.5)
	Employer/self-employed	1555 ( 7.5)
	Student	839 ( 4.1)
	Homemaker	3466 (16.7)
	Retired	745 ( 3.6)
	Unemployed	2825 (13.6)
Education (%)	High school or lower	5899 (28.5)
	College or higher	14813 (71.5)
Habitual alcohol intake (%)	Not at all	6969 (33.6)
	1 or less times per week	6699 (32.3)
Smoking status (%)	Non smoker	12267 (59.2)
	Current smoker	3628 (17.5)
	Former smoker	4817 (23.3)
Home ownership (%)	not having own house	6119 (29.5)
	having own house	14593 (70.5)
Number of COVID-19 vaccinations (%)	0	2457 (11.9)
	1	141 ( 0.7)
	2	15679 (75.7)
	3	2435 (11.8)
Severe psychological distress (K6 ≥13) (%)	No	18407 (88.9)
	Yes	2305 (11.1)
Adverse childhood experiences (%)	0	13309 (64.3)
	1 or more	7403 (35.7)
	2 or more times per week	7044 (34.0)
Subjective health status (%)	good	11647 (56.2)
	average	6253 (30.2)
	bad	2812 (13.6)
History of alcohol dependency (%)	No	20397 (98.5)
	Yes	315 ( 1.5)
History of depression (%)	No	18450 (89.1)
	Yes	2262 (10.9)
Household income (%)	Income < 4 million Japanese Yen	6204 (30.0)
	4 million ≤ income < 8 million	6486 (31.3)
	8 million ≤ income	3764 (18.2)
	Did not know/Did not want to answer	4258 (20.6)
Baseline FCV-19S total score (mean (SD))		17.01 (5.49)
FCV-19S total score one year later (mean (SD))		15.81 (5.52)
COVID-19 infection (%)	Not infected	16917 (81.7)
	COVID-19 not requiring oxygen therapy	3402 (16.4)
	COVID-19 requiring oxygen therapy	393 ( 1.9)

The coronavirus disease 2019 (COVID-19), The Fear of COVID-19 Scale (FCV-19S)

The predictors of changes in the FCV-19S total score over the course of 1 year from baseline based on the multiple regression analysis with IPW are shown in Table 2. The results indicated that the predictors of increase in FCV-19S scores were older age (estimate = 0.02, *p* < 0.001), male sex (estimate = 0.21, *p* = 0.0038), COVID-19 requiring oxygen therapy (estimate = 2.95, *p* < 0.001), higher baseline FCV-19S total score (estimate = 0.59, *p* < 0.001), severe psychological distress (estimate = 0.44, *p* < 0.001), never married (estimate = 0.45, *p* < 0.001), worsening subjective health status (good = reference; average, estimate = 0.59, *p* < 0.001; poor, estimate = 0.76, *p* < 0.001), a greater number of COVID-19 vaccinations (*n* = 0, reference; *n* = 1, estimate = 0.97, *p* = 0.0062; *n* = 2, estimate = 0.41, *p* < 0.001; *n* = 3, estimate = 0.49, *p* < 0.001), a history of alcohol dependency (estimate = 0.53, *p* = 0.026), and living with family members (estimate = 0.26, *p* = 0.0066). Conversely, the predictors of a decreased FCV-19S score included habitual alcohol intake (1 or fewer times per week, estimate = -0.25, *p* = 0.0007; 2 or more times per week, estimate = -0.19, *p* = 0.018), COVID-19 not requiring oxygen therapy (estimate = -0.99, *p* < 0.001), and a higher household income (income < 4 million Japanese yen = reference; 4 million ≤ income < 8 million, estimate = -0.20, *p* = 0.018; 8 million ≤ income, estimate = -0.45, *p* < 0.001). The predictors of changes in the FCV-19S total score determined without IPW are shown in Table S1. There was no multicollinearity among the independent variables used in the multiple regression analysis with or without IPW.

**Table 2:** Predictors of changes in Fear of COVID-19 Scale based on multiple regression analysis with inverse probability weighting

Factor	Group	Estimate	Standard Error	t value	*p* value
(Intercept)		4.19 (3.74- 4.64)	0.23	18.2	2E-73
Age		0.02 (0.01- 0.02)	0	6.72	1.8E-11
Sex	female	Reference			
	male	0.21 (0.07- 0.35)	0.07	2.89	0.0038
Living status	Living alone	Reference			
	Living with family member	0.26 (0.07- 0.45)	0.1	2.71	0.0066
Marital status	Married	Reference			
	Never married	0.45 (0.27- 0.64)	0.1	4.73	0.0000023
	Widowed	0.04 (-0.32- 0.40)	0.18	0.23	0.82
	Divorced	-0.02 ( -0.30- 0.26)	0.14	-0.14	0.89
Employment	Employee	Reference			
	Employer/self-employed	-0.21 (-0.44- 0.03)	0.12	-1.71	0.088
	Student	0.02 (-0.25- 0.28)	0.14	0.13	0.9
	Homemaker	0.04 (-0.17- 0.24)	0.1	0.34	0.74
	Retired	-0.21 (-0.57- 0.14)	0.18	-1.17	0.24
	Unemployed	-0.17 (-0.38- 0.04)	0.11	-1.62	0.11
Education	High school or lower	Reference			
	College or higher	-0.08 (-0.22- 0.05)	0.07	-1.17	0.24
Habitual alcohol intake	Not at all	Reference			
	1 or less times per week	-0.25 ( -0.40--0.11)	0.07	-3.39	0.0007
	2 or more times per week	-0.19 (-0.34--0.03)	0.08	-2.37	0.018
Smoking status	Non smoker	Reference			
	Current smoker	0.16 (-0.02- 0.33)	0.09	1.74	0.082
	Former smoker	-0.15 (-0.31- 0.02)	0.08	-1.78	0.075
Home ownership	not having own house	Reference			
	having own house	-0.05 (-0.19- 0.10)	0.08	-0.62	0.54
Number of COVID-19 vaccinations	0	Reference			
	1	0.97 (0.28- 1.67)	0.36	2.74	0.0062
	2	0.41 (0.22- 0.59)	0.1	4.25	0.000021
	3	0.49 (0.24- 0.74)	0.13	3.82	0.00013
Severe psychological distress (K6 ≥13)	No	Reference			
	Yes	0.44 (0.24- 0.64)	0.1	4.33	0.000015
Adverse childhood experiences	0	Reference			
	1 or more	-0.07 ( -0.20- 0.05)	0.07	-1.15	0.25
Subjective health status	good	Reference			
	average	0.59 (0.45- 0.72)	0.07	8.46	2.9E-17
	bad	0.76 (0.56- 0.95)	0.1	7.64	2.2E-14
History of alcohol dependency	No	Reference			
	Yes	0.53 (0.06- 1.00)	0.24	2.23	0.026
History of depression	No	Reference			
	Yes	-0.17 (-0.37- 0.03)	0.1	-1.67	0.094
Household income	Income < 4 million Japanese Yen	Reference			
	4 million ≤ income < 8 million	-0.20 (-0.36--0.03)	0.08	-2.36	0.018
	8 million ≤ income	-0.45 (-0.65--0.25)	0.1	-4.5	0.0000067
	Did not know/Did not want to answer	0.08 (-0.1- 0.25)	0.09	0.86	0.39
Baseline FCV-19S total score		0.59 (0.58- 0.60)	0.01	104.21	0
COVID-19 infection	Not infected	Reference			
	COVID-19 not requiring oxygen therapy	-0.99 (-1.15--0.83)	0.08	-12.28	1.5E-34
	COVID-19 requiring oxygen therapy	2.95 (2.53- 3.36)	0.21	14.05	1.2E-44

*p* < 0.05 was regarded as statistically significant using multiple regression analysis with forced entry

The coronavirus disease 2019 (COVID-19), The Fear of COVID-19 Scale (FCV-19S)

## Discussion

This study showed that the FCV-19S total score one year later decreased in comparison to that of the baseline survey conducted in 2022. Consequently, there is speculation that the initial fear associated with the COVID-19 pandemic, feared as an unknown infectious disease, has waned over time owing to the implementation of infection prevention measures, enhanced support systems, and the widespread distribution of vaccines. On the other hand, individuals with higher baseline FCV-19S total scores tended to have even higher scores one year later. This finding suggested that those more susceptible to fear may experience a worsening of fear over time. Therefore, we believe that it is necessary to adopt individualized approaches stratified by the severity of the infection when addressing the fear of COVID-19.

In our study, severe COVID-19 requiring oxygen therapy within one year from baseline was the most impactful predictor of increase in FCV-19S scores one year later. This finding aligns with those of prior research suggesting that individuals with severe COVID-19 are more prone to manifesting long-term psychological symptoms, commonly referred to as long COVID symptoms [28]. Conversely, participants who had relatively mild COVID-19 and did not need oxygen therapy had significantly lower FCV-19S scores than did their uninfected counterparts. This outcome can be explained from a resilience standpoint [29]. Despite facing the fear of contracting COVID-19, individuals who do not experience severe psychological trauma and successfully recover may develop resistance to the fear associated with COVID-19. Based on these results, it has been proposed that the severity of COVID-19 may warrant adjustments in subsequent psychological support.

Moreover, the number of administered COVID-19 vaccines was a predictor of changes in fear of COVID-19. Although Japan is recognized for its high vaccination rate compared to that of the global population [30], it is possible that vaccination is carried out passively, influenced by fear and societal pressure. COVID-19 vaccines have the potential to diminish the risk of contracting and developing severe COVID-19 [31]. However, adverse events such as anaphylactic shock have also been documented [32]. Previous research has indicated that trust in vaccines is correlated with an increased willingness to be vaccinated [33]. It is crucial for individuals to seek reliable information, fully comprehend the associated risks and benefits, and make informed decisions regarding the necessity of vaccination.

Interestingly, our investigation revealed an inverse correlation between baseline income levels and changes in FCV-19S scores. Previous reports have suggested that the behavioral constraints imposed during the COVID-19 pandemic contributed to economic stagnation and increased societal poverty. Against the backdrop of the COVID-19 pandemic, characterized by an increasing prominence of mental health issues among citizens, individuals with lower incomes experienced more pronounced psychological distress [34]. Additionally, low income is not only a contributing factor to deteriorating mental health but is also linked to various socioeconomic vulnerabilities, including unemployment and housing instability [35]. Despite the Japanese government's efforts to provide one-time cash payments to support households, reports suggest that transient economic interventions may not substantially alter consumption behavior in contexts marked by heightened COVID-19-related anxiety and that their impacts may be constrained [12]. Therefore, we believe that it is crucial to establish a robust system ensuring stable employment, thereby empowering individuals to strategize for their future well-being from both mental health and socioeconomic perspectives.

Furthermore, habitual alcohol intake, compared to abstaining from alcohol entirely, was a predictor of decrease in FCV-19S scores. Previous research has indicated that moderate alcohol consumption has a calming effect on anxiety and depression [36]. Therefore, in the context of the unstable societal situation with the spread of emerging infectious diseases, moderate alcohol consumption was suggested to have a protective effect on mental health. On the other hand, a history of alcohol dependence was identified as a predictor of increase in FCV-19S scores. Alcohol dependence is often comorbid with other mental disorders, including depression, making it a major public health concern [37]. In fact, in this study, the presence of severe psychological distress emerged as a predictor of increase in FCV-19S scores. Lockdowns during the COVID-19 pandemic have been identified as a factor causing psychological distress, and further mental and physical issues may have emerged as a result of individuals attempting to self-treat this distress through excessive alcohol consumption [38]. Considering the risks and benefits of alcohol consumption, it might be advisable to refrain from drinking with the intention of alleviating the fear of COVID-19.

Notably, having never been married, compared to being married, was a statistically significant predictor of an increase in FCV-19S score. According to previous research, social isolation is known to be a risk factor for depression [39], and this trend was reported to persist during the COVID-19 pandemic [40]. Therefore, especially in situations where behavior is restricted, it was estimated that the increase in fear of COVID-19 for individuals in social isolation can be somewhat prevented by actively establishing support systems, including the use of online services [41]. On the other hand, cohabitation with family members was a predictor of increase in FCV-19S scores. This result contrasts with the expectation that living with family members would reduce the risk of social isolation and lead to decreased fear of COVID-19. Previous research has suggested that individuals may experience greater distress concerning their family members potentially contracting COVID-19 (or inadvertent transmission to others) than about their own infection [42]. Additionally, studies have reported heightened psychological distress among individuals cohabiting with elderly or handicapped family members during the COVID-19 pandemic [43]. Hence, the presence of vulnerable cohabiting family members who are susceptible to infection may indirectly contribute to an increased fear of COVID-19.

In this study, sex differences also emerged as predictors of changes in the FCV-19S score. Notably, in contrast to female sex, male sex was a predictor of increase in FCV-19S scores one year later. Intriguingly, certain prior studies have indicated a tendency for women to experience more pronounced fear of COVID-19 than men [44]. Furthermore, the impact of lockdown measures during the COVID-19 pandemic has been recognized as potentially rendering women more vulnerable to feelings of social isolation than men [45]. However, with the progression of COVID-19 countermeasures and the resumption of social interactions, the stress coping style of women may effectively function as a form of resilience against the fear of COVID-19 [46]. Notably, the societal conditions in this study, occurring 2–3 years after the onset of the pandemic, resulted in advancements in preventive measures and social support compared to those observed during the period when previous studies were conducted. These shifts in societal background could account for the discrepancies in results pertaining to sex differences in FCV-19S scores compared to earlier research.

Our study has several limitations. First, this study was conducted with a primary focus on Japanese residents, making it uncertain whether the findings can be extrapolated to other countries and cultures. Second, our reliance on internet-based surveys may have introduced selection bias into our data since low-income people or elderly people could be excluded from the data collection due to poor web access. Third, despite our efforts to account for potential confounding variables, the presence of unidentified or residual confounders could have influenced our study outcomes. Fourth, the independent variables used in this study were mainly general sociodemographic characteristics, which limits their applicability from a clinical perspective. We refrained from gathering detailed clinical characteristics due to feasibility concerns about the survey. Fifth, the presence or absence of oxygen therapy is an important factor in the severity classification of COVID-19 [47], but information on the level of care intensity needed was lacking in our study. Therefore, it is considered necessary to conduct surveys targeting medical institutions and to examine patient backgrounds in a clinical context, including post-COVID-19 rehabilitation. Finally, while our study has the epidemiological strength of a large sample size, there are limitations in its clinical impact. Thus, we aimed to conduct further research based on this study to investigate the clinical factors influencing the fear of COVID-19 and their causal relationships.

## Conclusion

During the observation period, the FCV-19S scores significantly decreased. This was considered influenced by the implementation of measures to prevent COVID-19 and the enhancement of support systems. Contracting severe COVID-19 requiring oxygen therapy within one year of baseline was the most impactful predictor of an increase in the FCV-19S score. This finding suggested the possibility of a prolonged trauma response to a severe physical crisis. On the other hand, we found that mild COVID-19 not requiring oxygen therapy was a predictor of a decrease in FCV-19S score. Therefore, we believe that it is necessary to adopt individualized approaches stratified by the severity of the infection when addressing the fear of COVID-19.

### Supplementary Information

Supplementary Material 1.

## Data Availability

The data used in this study are not available in a public repository because they contain personally identifiable or potentially sensitive patient information. Based on the regulations for ethical guidelines in Japan, the Research Ethics Committee of the Osaka International Cancer Institute imposed restrictions on the dissemination of the data collected in this study. To request data, please contact the institutional review board of the ethics committee. The contact information for our ethics committee is as follows: the Research Ethics Committee of the Osaka International Cancer Institute; Chuo-ku, Otemae 3–1-69, Osaka, Japan, Postal Code 541–8567, Phone + 81–6-6945–1181.

## Abbreviations

COVID-19: The coronavirus disease 2019
FCV-19S: The Fear of COVID-19 Scale
JASTIS: Japan “Society and New Tobacco” Internet Survey
